# SPECT Imaging with Tc-99m-Labeled HYNIC-FAPI-04 to Extend the Differential Time Window in Evaluating Tumor Fibrosis

**DOI:** 10.3390/ph16030423

**Published:** 2023-03-10

**Authors:** Xiu Luo, Zhe Zhang, Chao Cheng, Tao Wang, Danzhou Fang, Changjing Zuo, Gengbiao Yuan, Rou Li, Xiao Li

**Affiliations:** 1 Department of Nuclear Medicine, Shanghai Changhai Hospital, Shanghai 200433, Chinalixiao_nm@smmu.edu.cn (X.L.); 2 Department of Nuclear Medicine, The Second Affiliated Hospital of Chongqing Medical University, Chongqing 400010, China

**Keywords:** ^99m^Tc-HYNIC-FAPI-04, differential time window, tumor fibrosis, fibroblast activating protein, FAP inhibitor, FAPI-04

## Abstract

The so-far used Ga-68- or F-18-labelled tracers are of a relative short time window in differentiating tumor fibrosis. SPECT applicable imaging probe, ^99m^Tc-HYNIC-FAPI-04, was synthesized and evaluated in tumor cells and animal models of FAP-positive glioma and FAP-negative hepatoma, and then compared with ^18^F-FDG or ^68^Ga-FAPI-04 PET/CT. The radio-labeling rate of ^99m^Tc-HYNIC-FAPI-04 was greater than 90%, and the radiochemical purity was >99% after purification with sep-pak C18 column. In vitro cell uptake experiments of ^99m^Tc-HYNIC-FAPI-04 showed good FAP binding specificity, and the cellular uptake significantly decreased when blocked by DOTA-FAPI-04, reflecting the similar targeting mechanism of HYNIC-FAPI-04 and DOTA-FAPI-04. SPECT/CT imaging showed that U87MG tumor was distinguishable and of a high uptake of ^99m^Tc-HYNIC-FAPI-04 (2.67 ± 0.35 %ID/mL at 1.5 h post injection (h P.I.), while tumor signal of FAP-negative HUH-7 was as low as 0.34 ± 0.06 %ID/mL. At 5 h P.I., U87MG tumor was still distinguishable (1.81 ± 0.20 %ID/mL). In comparison, although U87MG tumor was of obvious ^68^Ga-FAPI-04 uptake and clearly visible at 1 h P.I., the tumorous radioactive signals were fuzzy at 1.5 h P.I. ^99m^Tc-HYNIC-FAPI-04 specifically bound to FAP-positive tumors and qualified with the ability of evaluating tumor fibrosis over longer time windows.

## 1. Introduction

Fibroblast activating protein (FAP) is a type II transmembrane serine protease that is highly expressed as a dimer on the surface and matrix of up to 90% of epithelial cancer tumor-associated fibroblasts (CAFs) [1,2], especially in breast cancer, colorectal cancer, pancreatic cancer, cervical cancer and other malignant tumors characterized by proliferative hyperplasia reaction of connective tissue [3]. The high expression of FAP in tumor interstitium and the poor prognosis of tumor patients suggests that FAP is a key factor in tumor growth, proliferation, invasion and metastasis [4,5], which further makes FAP the most prominent target for tumor diagnosis and treatment in the field of nuclear medicine. FAP inhibitor (FAPI)-based imaging is even expected to replace the most widely used radioactive diagnostic tracer FDG in clinical practice, due to the convenience in simplifying the integration of diagnosis and treatment via changing nuclides. FAP plays a complex role in the regulation of extracellular matrix [6], and its related specific inhibitors have been developed in different isomers [7,8,9]. Recent studies have shown that quinoline-based FAP inhibitors have more prominent specificity in targeting tumors [10]. Among them, FAPI-04 showed stronger binding efficiency and better biochemical kinetic properties, as it was able to in vivo combine with FAP in 10 min after injection, with low non-targeted soft tissue background, and was completely cleared by the kidneys at 3 h post injection (P.I.) [10,11].

^68^Ga-labeled FAPI-04 for positron emission tomography/computed tomography (PET/CT) or PET/magnetic resonance imaging (MRI) can reflect the tumor profile, such as types and microenvironment of tumor cases, and show a high contrast between the tumor and healthy tissue [12]. In PET imaging, ^68^Ga-FAPI-04 showed superior detection performance compared to ^18^F-FDG in pancreatic, gastric, and lung cancers [13,14]. In addition to ^68^Ga-FAPI-04, radiopharmaceuticals such as ^18^F-, ^64^Cu-, ^90^Y-, ^177^Lu- and ^225^Ac-labeled FAPI-04 have been used for PET imaging or selective internal irradiation [15,16,17,18]. Despite the increasing use of positron radionuclides in clinical medicine, PET scans are somewhat costly, and PET devices are not widely available, especially in the low-income or under-developed areas. Moreover, the half-life of commonly-used positron radionuclides (Ga-68 and F-18) is short, so the differential time window (dT), that is to say the time length of imaging modality with differential diagnosis capability, is too short to do some time-consuming examinations, such as the delayed scan and dual-phase scan. Except for the PET nuclides, ^99m^Tc is one of the most commonly used nuclides in clinic because of its suitable half-life (t_1/2_ = 6.02 h), good radiophysical properties (E_γ_ = 141 keV), low cost and easy availability [19]. ^99m^Tc-radiolabeled compounds are commonly used in single photon emission computed tomography (SPECT) imaging, correspondingly, 6-hydrazinonicotinate-aminocaproic (HYNIC) often acts as a bifunctional chelating ligand to conjugate ^99m^Tc to various biomolecules, including antibodies and peptides [20,21,22]. For instance, ^99m^Tc-HYNIC-PSMA has been reported in clinical studies and has shown high uptake in PSMA-positive lesions in patients with prostate cancer [23,24], making it an alternative method for PSMA-specific diagnosis.

Therefore, in this study, based on the developed synthesis of ^99m^Tc-HYNIC-FAPI-04 [25,26], the feasibility in diagnosing FAP was evaluated, especially focusing on the extension of differential time window. In the additional comparison with ^68^Ga-FAPI-04 PET or ^18^F-FDG PET, the imaging performance of ^99m^Tc-HYNIC-FAPI-04 SPECT was evaluated in mouse xenografted with U87MG-based FAP-positive tumors.

## 2. Results

### 2.1. Radiolabeling and Stability

**Figure 1:**
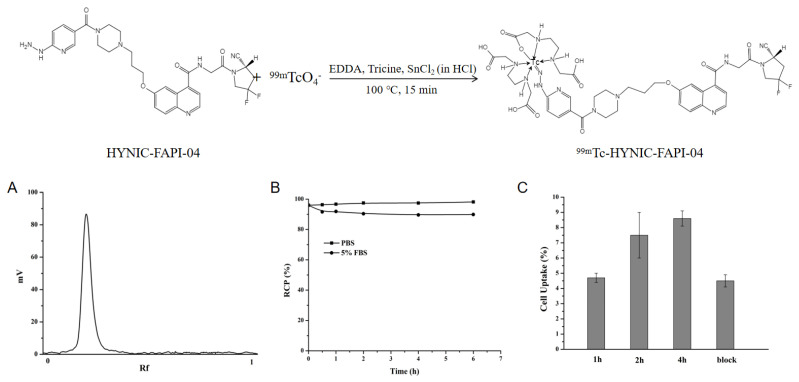
Schematic preparation of ^99m^Tc-HYNIC-FAPI-04, and the in vitro characteristics, including (**A**) the labeling rate, (**B**) time-dependent stability in PBS and 5% FBS, and (**C**) U87MG-specific cellular binding efficiency (EDDA: ethylenediamine diacetic acid, PBS: phosphate buffer solution, FBS: fetal bovine serum).

The labeling process of ^99m^Tc-HYNIC-FAPI-04 is shown in Figure 1. HYNIC-FAPI-04 was labeled with Na^99m^TcO_4_ using SnCl_2_ in diluted HCl as the reducing agent, and ethylenediamine diacetic acid (EDDA) and Tricine served as co-ligands to obtain ^99m^Tc-HYNIC-FAPI-04. ^99m^Tc-HYNIC-FAPI-04 was primarily obtained with a 15-min reaction at 100 °C, and quality control was carried out through instant thin layer chromatography (iTLC) with thin layer chromatography paper as stationary phase and acetone as mobile phase. The labeling rate was normally higher than 90%. After purification with C18 column, the radiochemical purity (RCP) of ^99m^Tc-HYNIC-FAPI-04 was nearly 100% (Figure 1A).

As shown in Figure 1B, ^99m^Tc-HYNIC-FAPI-04 was stable in phosphate buffer solution (PBS) and 5% fetal bovine serum (FBS) solution at room temperature for 6 h, and the stability was better in PBS, meeting the requirements for storage and delivery as a daily-prepared radiopharmaceutical.

### 2.2. In Vitro Binding Ability

The results of cellular binding experiment of ^99m^Tc-HYNIC-FAPI-04 are shown in Figure 1C. The ^99m^Tc-HYNIC-FAPI-04 uptake of U87MG cells (FAPI+) increased with the extension of incubation time and the binding rate reached 8.6 ± 0.5% after 4 h incubation, manifesting the necessity of a longer interaction between tracer and target. In vitro cell binding competitive assay results obtained by adding 250 times excess unlabeled DOTA-FAPI-04 as a competitive agent showed that the binding of ^99m^Tc-HYNIC-FAPI-04 to U87MG cells was significantly reduced (8.6 ± 0.5% vs. 4.5 ± 0.4%, *p*-value < 0.05) and of a same target with DOTA-FAPI-04.

### 2.3. SPECT/CT Imaging with ^99m^Tc-HYNIC-FAPI-04

**Figure 2:**
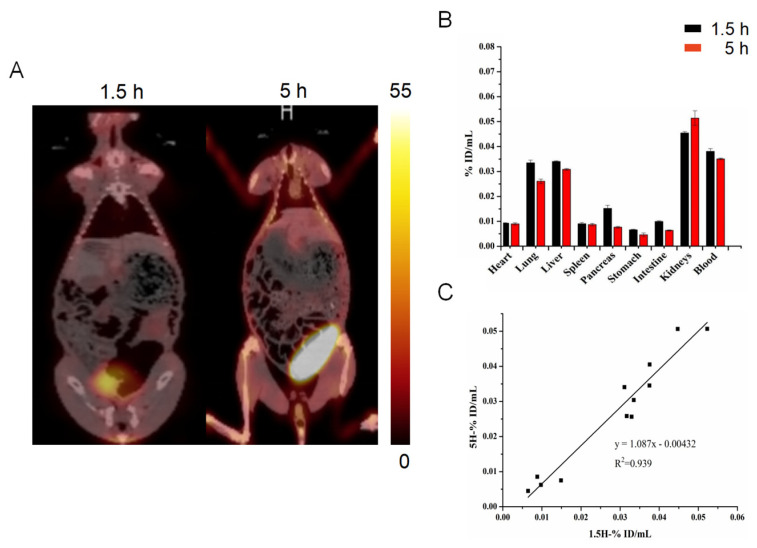
^99m^Tc-HYNIC-FAPI-04 SPECT images of rabbits at different time points. The coronal whole-body images at 1.5 h P.I. and 5 h P.I. (**A**) and radioactive counts (presented as %ID/mL) of ROIs on SPECT at 1.5 h P.I. and 5 h P.I. (**B**). The linear regression between radioactive counts of ROIs at 1.5 h P.I. and 5 h P.I. (these dots represented the ROIs above) (**C**).

The time-dependent in vivo distribution of ^99m^Tc-HYNIC-FAPI-04 was firstly explored in rabbits. As shown in Figure 2A, most organs were of low uptake of ^99m^Tc-HYNIC-FAPI-04 at 1.5 h P.I. and 5 h P.I., providing a clean imaging background and target to background ratio of lesion. Compared with other organs, the liver (0.03 ± 0.00 %ID/mL) and kidneys (0.04 ± 0.00 %ID/mL) were of relative higher tracer uptake at 1.5 h P.I., which indicated radioactive tracers were mainly excreted through the hepatoenteric metabolism and urinary system metabolism. At 5 h P.I., the in vivo distribution pattern of ^99m^Tc-HYNIC-FAPI-04 was consistent with that at 1.5 h P.I.; in addition, the organ-specific uptake between 1.5 h P.I. and 5 h P.I. was of a high repeatability with a linearly dependent coefficient of 0.939, meaning the feasibility of a relatively longer scanning time window of ^99m^Tc-HYNIC-FAPI-04 SPECT/CT.

**Figure 3:**
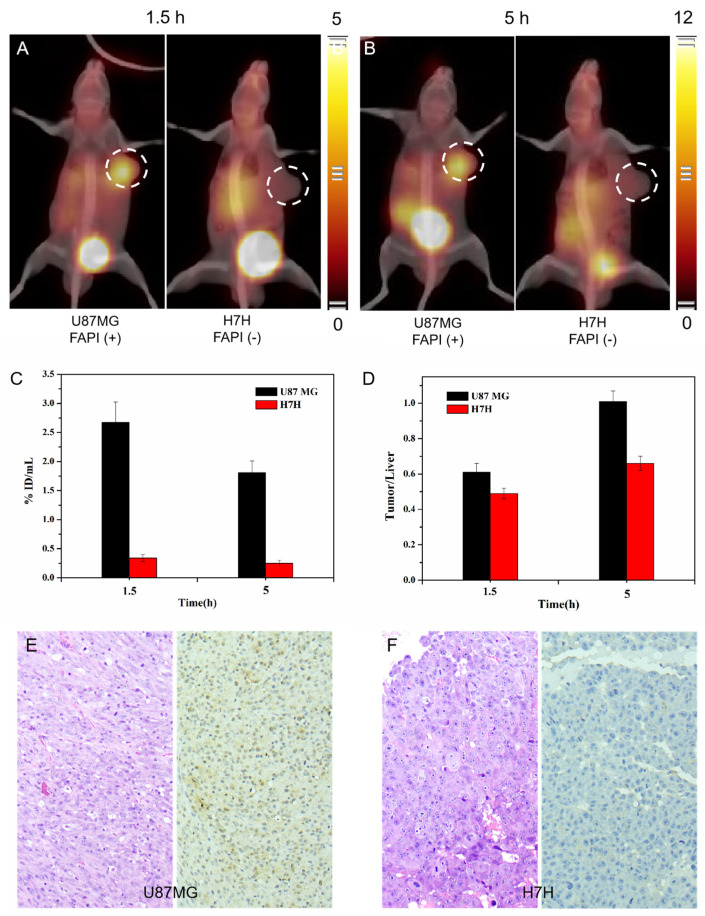
^99m^Tc-HYNIC-FAPI-04 SPECT images of mice models with FAP-positive or FAP-negative xenografts at 1.5 h P.I. (**A**) and 5 h P.I. (**B**). The radioactive counts of FAP-positive or FAP-negative xenografts (**C**) and the ratios of xenografts to liver (**D**). The corresponding HE staining and quantitative analysis with immunohistochemical proves of FAP-positive (**E**) or FAP-negative tumors (**F**).

3D whole-body ^99m^Tc-HYNIC-FAPI-04 SPECT/CT images were obtained in severely immunodeficient NOD SCID mice bearing U87MG (FAPI+) or HUH-7 (FAPI-) tumor xenografts. As shown in Figure 3, at 1.5 h P.I., ^99m^Tc-HYNIC-FAPI-04 uptake was clearly observed in FAP-positive U87MG tumor at 2.67 ± 0.35 %ID/mL. In contrast, uptake in FAP-negative HUH-7 xenografts was as low as 0.34 ± 0.06 %ID/mL, and no differentiable signal was observed when compared with background. At 5 h P.I., U87MG tumor was still of a high uptake at 1.81 ± 0.20 %ID/mL, an uptake value only decreased by half when compared with 1.5 h, indicating the stable binding of ^99m^Tc-HYNIC-FAPI-04 to FAP in U87MG tumor. The tumor status can be therefore observed for a relatively long time of up to 5 h, especially given that there were higher tumor to liver ratios in the late phase of SPECT/CT examination.

In addition, the relative high uptake was clearly observed in the liver, kidneys and bladder at 1.5 h P.I., and a high uptake in intestinal tracts emerged at 5 h P.I., suggesting that radioactive tracers were mainly excreted through the hepatoenteric metabolism and urinary system metabolism.

### 2.4. Comparison with Other Imaging Modalities

#### 2.4.1. Comparison with ^18^F-FDG PET

**Figure 4:**
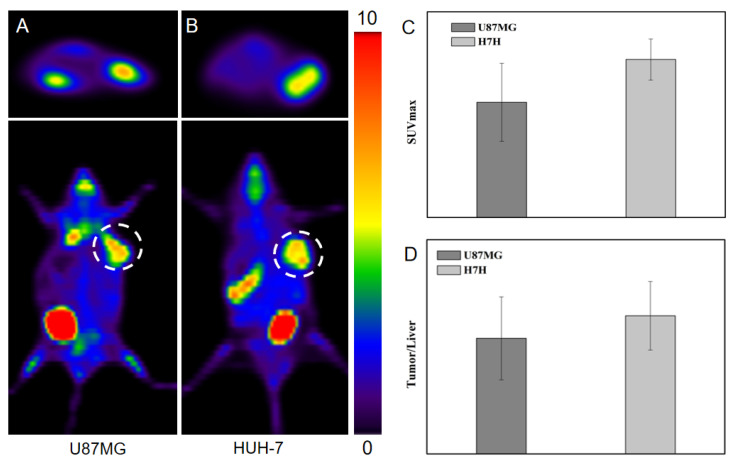
FDG PET of mice models of (**A**) U87MG and (**B**) HUH-7, and (**C**) the corresponding quantitative analysis, such as SUV_max_ and (**D**) tumor to liver ratio of SUV_max_. The white dotted circles marked the FAP-positive or FAP-negative tumors.

To perform a proof-of-concept study and evaluate the tracer performance of ^18^F-FDG compared to ^99m^Tc-HYNIC-FAPI-04, PET/CT studies were performed using xenografts U87MG and HUH-7. Measurements at 60 min P.I. showed comparable biodistribution (Figure 4A,B) and specific uptake of ^18^F-FDG in tumors containing U87MG and HUH-7. Both U87MG and HUH-7 tumors showed obvious radioactive signals, and there was no significant difference between the target ratio and the tumor–liver ratio, indicating that tumor fibrosis was not the fundamental factor in tumor metabolism. In other words, FAP imaging can provide extra information in uncovering the subtle differences, so as to facilitate the personalized medicine. For example, the comparable tumor to liver ratios of U87MG (2.16 ± 0.78) and HUH-7 (2.58 ± 0.64) in FDG PET was adverse in making a judgement on primary lesions that were potentially originated from glioma or hepatoma, but ^99m^Tc-HYNIC-FAPI-04 SPECT/CT at 5 h P.I. showed feasibility in differentiating the difference (Figure 3D).

#### 2.4.2. Comparison with ^68^Ga-FAPI-04 PET

**Figure 5:**
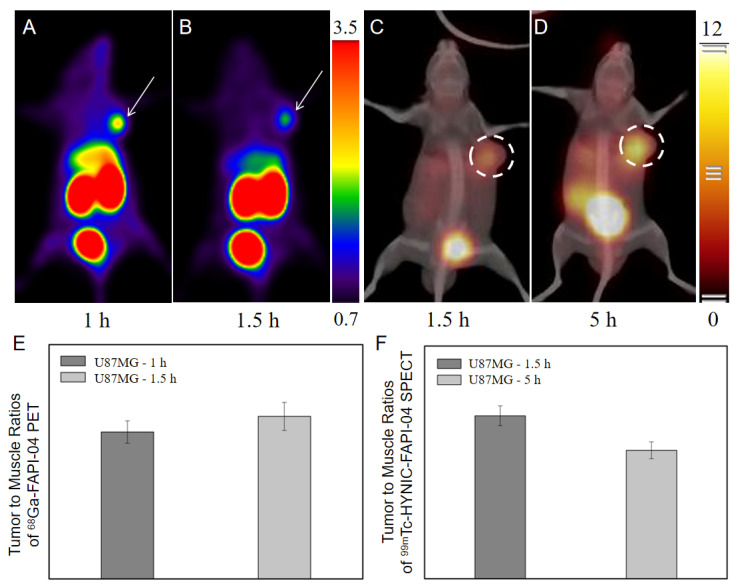
^68^Ga-FAPI-04 PET images of FAP-positive tumors at different time point (**A**,**B**) and ^99m^Tc-HYNIC-FAPI-04 SPECT images of FAP-positive tumors at different time point (**C**,**D**). The comparison of ratios of tumor to muscle between 1.5 h P.I. and 5 h P.I. in ^68^Ga-FAPI-04 PET (**E**) and ^99m^Tc-HYNIC-FAPI-04 SPECT (**F**). The white arrow and white dotted circles all pointed to FAP-positive tumors.

As the comparison exhibits in Figure 5, for the distinguishable SPECT/CT images of U87MG tumor acquired at 1.5 h P.I., although U87MG tumor was of obvious ^68^Ga-FAPI-04 uptake (SUV_max_ = 0.362 ± 0.069) and clearly visible at 1 h P.I., the tumorous radioactive signals tended to be fuzzy at 1.5 h P.I. (SUV_max_ = 0.286 ± 0.033). At 5 h P.I., U87MG tumor was still distinguishable with tracer uptake as high as 1.81 ± 0.20 %ID/mL.

In the comparison of extending the differential time window (Figure 5C), tumor to muscle ratios were maintained in a comparable level when compared with the ones of routine imaging, but the proved effective dT extended from 1.5 h P.I. of ^68^Ga-FAPI-04 PET to 5 h P.I. of ^99m^Tc-HYNIC-FAPI-04 SPECT, when utilizing the similar FAPI-04 analogs. Although 1.5 h P.I. was a shared optimal imaging time point of ^68^Ga-FAPI-04 PET and ^99m^Tc-HYNIC-FAPI-04 SPECT, the increased tumor to background at 5 h P.I. of ^99m^Tc-HYNIC-FAPI-04 SPECT was of clinical benefits to find metastases with low tracer uptake.

### 2.5. Validation of Fibrosis with a Longer Differential Time Window

**Figure 6:**
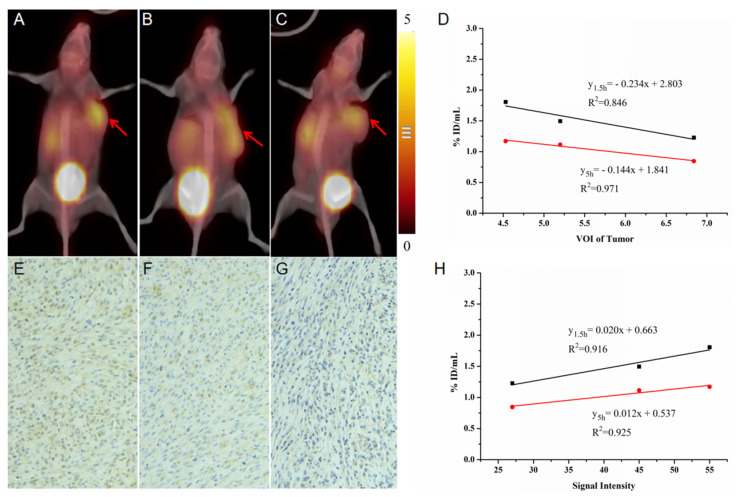
The correlation between ^99m^Tc-HYNIC-FAPI-04 SPECT and tumor progression, including tumor volume and FAP expression quantified by signal intensity of immunohistochemical (IHC). The volume progression of tumor and corresponding FAP expression in IHC (**A**–**G**), and correlation analysis result (**D**). Correlation between tracer uptake on ^99m^Tc-HYNIC-FAPI-04 SPECT and FAP expression (**H**). The red arrow pointed to FAP-positive tumors.

Figure 6 summarizes the correlation between ^99m^Tc-HYNIC-FAPI-04 SPECT and tumor progression, including tumor volume and FAP expression. The tracer uptake characterized as %ID/mL significantly correlated with tumor progression. For tumor volume, a negative correlation was detected that resulted in the delay of fibrosis in the rapid formation of tumor matrix (y_1.5h_ = −0.234x + 2.803 and y_5h_ = −0.144x + 1.841); accordingly, a positive correlation was undoubtedly manifested between tracer uptake and FAP expression (y_1.5h_ = 0.020x + 0.663 and y_5h_ = 0.012x + 0.537).

Notably, the tracer uptake at 5 h post injection (R^2^_tumor volume_ = 0.971 and R^2^_FAP expression_ = 0.925) was of a higher correlation coefficient than the quantification acquired at 1.5 h post injection (R^2^_tumor volume_ = 0.846 and R^2^_FAP expression_ = 0.916). The total clearance of blood background at a later time point contributed to the increase of correlation. In clinical practice, due to the existence of abundant new vessels in tumor, a correlation that avoided the interference from blood background was meaningful to FAP imaging.

## 3. Discussion

In clinic, the diagnostic imaging techniques of cancers include CT, MRI, ultrasound and nuclear medicine. Among the molecular imaging modalities, nuclear medicine is more sensitive to biochemical changes, and relatively bio-safe to patients, meanwhile, the biochemical characteristics of tumors can be observed at the molecular level [27,28]. Although ^18^F-FDG is an already-used PET/CT tracer of glucose metabolism that can be significantly taken up by hypermetabolic malignancies [29], ^18^F-FDG can also concentrate in inflammatory cells, causing false positive diagnosis [30]. A technique with more specificity for tumor imaging is of more potential benefits to patients. For the essential interstitial components throughout the whole process of tumor genesis and proliferation, FAP-related indicators are potentially more stable and reliable in developing a molecular imaging protocol than microenvironment-based ones, such as immunoPET. Hence, the development of Tc-99m-labeled targeted tracers is meaningful to expand the clinical applications of nuclear medicine, especially those targeted therapeutics on the basis of widely expressed targets, such as FAP.

Given the fact that tumor is composed of tumor cells and tumor matrix, as well as that FAP is highly expressed in the most abundant CAF [31,32], hence, a large number of FAP inhibitors that bind specifically to FAP have been developed [9], among which FAPI-04 has been extensively studied for its good tumor targeting effect and already utilized in diagnosing tens of cancers [25]. As an analog of DOTA-FAPI-04 and proved in this research, ^99m^Tc-HYNIC-FAPI-04 SPECT/CT was sensitive to tumor progression, such as tumorous volume and FAP expression (Figure 6), providing a monitoring method for dynamic changes in tumor progression. ^99m^Tc-HYNIC-FAPI-04 showed the replaceable role in vitro and in vivo via binding FAP to CAFs (Figure 1 and Figure 2), together with the facts that SPECT/CT is more popular than PET/CT and of recent advances in quantification and resolutions; hence, ^99m^Tc-HYNIC-FAPI-04 SPECT/CT is of a promising role in diagnosing fibroblasts.

In practice, the necessity of long time and dynamic observation on FAP expression, especially on the tumor-associated fibroblasts, stands to reason due to the proven relationship between FAP and prognosis, as well as the development of dual-phase examinations. Therefore, the extension of differential time window from tens of minutes to no less than 5 h was the first concern and the primary achievement in this research.

As dT was determined by the affinity between target and targeting molecule as well as the radio-physical of nuclides, Tc-99m was an optimal supporter of dual-phase imaging or molecular imaging modalities that need a relatively long time of metabolism. For example, Technetium-99m methoxyisobutyronitrile (^99m^Tc-MIBI) two-phase scintillate imaging is the primary preoperative localization method for patients with primary hyperparathyroidism or secondary hyperparathyroidism [33,34]. Moreover, cardiac imaging using technetium pyrophosphate (^99m^Tc-PYP) can sensitively and specifically distinguish cardiac amyloidosis between light-chain and the transthyretin cardiac amyloidoses in patients with advanced disease [35]. More recently, ^99m^Tc-PSMA SPECT has been extensively used as a supplement of personalized imaging, and the differential value was comparable to ^68^Ga-PSMA-11 PET, particularly in the cohorts with PSA higher than 2.10 ng/mL [36]. In consequence, ^99m^Tc-HYNIC-FAPI-04 SPECT was also of potential in exploring more capacities that were not qualified by FAPI-04 PET.

Besides these proved possibilities, there were also some limitations in this research. More type of tumors with high FAP expressed should be used for validation of ^99m^Tc-HYNIC-FAPI-04 SPECT imaging, such as the tumor model co-cultured with tumor matrix. Further studies dedicated to evaluating the diagnostic performance in tumor fibrosis are highly warranted.

## 4. Materials and Methods

### 4.1. Reagents and Equipment

All chemical reagents and solvents were purchased from Merck Sigma-Aldrich Co., Ltd. (Shanghai, China), and can be used without further purification. The Dulbecco′s modified Eagle′s medium (DMEM) and FBS were purchased from Gibco Life Technology Co., Ltd. (New York, NY, USA). The precursors of FAPI-04 was purchased from Shanghai Nice-labeling Biotechnology Co., Ltd. (Shanghai, China), and HYNIC-FAPI-04 was obtained from Shanghai Junna Medical Technology Co., Ltd. (Shanghai, China). Na^99m^TcO_4_ was freshly eluted with sodium chloride through a ^99^Mo/^99m^Tc generator obtained from Shanghai Atom Kexing Pharmaceutical Co., Ltd. (Shanghai, China). ^18^F-FDG was purchased from Shanghai Atom Kexing Pharmaceutical Co., Ltd.

The radioactive thin layer chromatographic instrument Mini-Scan (Eckert & Ziegler, Bioscan Inc., Poway, CA, USA) was used for quality control, and a PET/CT scanner (Siemens Healthcare, Erlangen, Germany) and SPECT/CT scanner (Symbia T16, Siemens, Germany) were used for animal imaging.

### 4.2. Radiopharmaceuticals and Quality Control

A kit was developed for convenient used in radiolabeling, which contained 20 µg HYNIC-FAPI-04, 10 mg EDDA and 20 mg tricine. For radiolabeling, 50 μL SnCl_2_ solution (1 mg/mL in 0.05 M HCl) was added to the kit. Then, 5 mCi (185 MBq) of eluted Na^99m^TcO_4_ was added immediately. The mixture was heated for 15 min at 100 °C to prepare the raw product of ^99m^Tc-HYNIC-FAPI-04, which was then purified with sep-pak C-18 column. 50% alcohol and normal saline were successively used as the flow separation phase.

The radiochemical purity of ^99m^Tc-HYNIC-FAPI-04 was determined by iTLC. The amount of ^99m^Tc-HYNIC-FAPI-04 (R_f_ = 0) and free ^99m^TcO_4_^−^ (R_f_ = 1) were determined using thin layer chromatography paper as stationary phase and acetone as mobile phase. 

The stability of ^99m^Tc-HYNIC-FAPI-04 in vitro was tested in phosphate buffer saline (0.01 M, pH = 7.4) and 5% FBS. ^99m^Tc-HYNIC-FAPI-04 was mixed with PBS or 5% FBS and kept at room temperature for 6 h. At different time points (0.5, 1, 2, 4, 6 h), the RCP of ^99m^Tc-HYNIC-FAPI-04 in PBS and 5% FBS was determined by the above iTLC method.

The preparation of ^68^Ga-FAPI-04 was reported in previous work [37]. The rinsed ^68^Ga from the ^68^Ge/^68^Ga generator (ITM, Munich, Germany) was mixed with the precursor FAPI-04 in 0.25 M sodium acetate and reacted at 100 °C for 10 min. ^68^Ga-FAPI-04 was obtained by coupling ^68^Ga with the DOTA of FAPI-04 and of an RCP > 95%.

### 4.3. Cell Culture and In Vitro Binding Efficiency

U87MG (human glioma cells) and HUH-7 cells were purchased from the Chinese Infrastructure of Cell Line Resource. U87MG and HUH-7 cells in DMEM with 10% FBS and double antibody were cultured in an incubator containing 5% CO_2_ at 37 °C.

In vitro cell uptake of ^99m^Tc-HYNIC-FAPI-04 was performed with U87MG cells. About 3 × 10^5^ cells were inoculated in 24-well cell culture plates (five multiple pores in each group) and then incubated with ^99m^Tc-HYNIC-FAPI-04 and kept at 37 °C for 1, 2 and 4 h. To determine the specific cell uptake, a blocking assay was set up to block U87MG cells with the analogous precursor of FAPI-04. At each incubation end, the medium was removed, and the cells were washed with saline solution. Cells were finally cleaved with 1 M NaOH, and the radioactive counts were measured using a gamma counter.

### 4.4. Animal Models

Animal studies were approved by Shanghai Changhai Hospital Ethics Committee (Shanghai, China; approval number: CHEC2021-071) and carried out in accordance with the principles of laboratory animal care. Severely immunodeficient male NOD SCID mice were obtained from Beijing Vital River Laboratory Animal Technology Co., Ltd. (Beijing, China). In order to establish U87MG subcutaneous tumor model, U87MG cells (4 × 10^6^ in 100 μL DMEM) were inoculated into the right anterior axilla. When the diameter reaches about 5~7 mm, tumor can be used to study the biological distribution. HUH-7 subcutaneous tumor models were obtained following the same protocol. 5 mice in each group. Five male New Zealand white rabbits (9–12 weeks of age, weighing 2.0–2.5 kg) were purchased from Shanghai Sippr-BK LAB Animal Co., Ltd. (Shanghai China) to explore the patterns of in vivo distribution and metabolism of ^99m^Tc-HYNIC-FAPI-04.

### 4.5. SPECT/CT Imaging

SPECT/CT imaging was performed in mice carrying U87MG glioma or HUH-7 hepatocellular carcinoma xenografts. After anaesthesia, 7.4 MBq ^99m^Tc-HYNIC-FAPI-04 for each mouse and 111 MBq ^99m^Tc-HYNIC-FAPI-04 for each rabbit were injected into the caudal vein. Imaging was performed with a low-energy, high-resolution SPECT/CT scanner (frame time, 25 s; slice, 0.75 mm) at 1.5 and 5 h after injection.

### 4.6. PET/CT Imaging

For mice, ^68^Ga-FAPI-04 PET and ^18^F-FDG PET were performed immediately after SPECT scans. Mice were kept fasting for 8 h before the scans. For ^18^F-FDG PET, mice were anesthetized and each was injected with 7.4 MBq ^18^F-FDG to the tail vein. PET/CT imaging in mice with glioma and liver cancer was performed at 1 h post injection. The mice were fixed on the examination bed in a prone position for CT scanning (effective current, 170 mAs; voltage, 120 kV; slice, 0.75 mm) and PET collection.

^68^Ga-FAPI-04 PET/CT scans were performed at 60 min and 90 min post injection. Mice were injected with ^68^Ga-FAPI-04 to the tail vein at 7.4 MBq/mouse. The mice were scanned with the same protocol with ^18^F-FDG PET.

### 4.7. Definition and Quantification of ROIs

On the PET/CT image, SUV was calculated by drawing the circular areas of interest, including the tumor, liver and muscle, and then the SUV_max_ was determined. The ratio of tumor to muscle and the ratio of tumor to liver were calculated. On the SPECT/CT image, for mice, the tumor, liver, and total body areas of interest were mapped to obtain the total counts and volume, and then the %ID/mL of tumor and liver were calculated. Similarly, the areas of interest of rabbits were manually drawn on the SPECT/CT image to obtain the counts and volume, and the organ-specific uptakes of ^99m^Tc-HYNIC-FAPI-04 were quantified as %ID/mL.

### 4.8. Immunohistochemistry and Quantification

For each xenograft that was harvested immediately after the imaging, hematoxylin-eosin staining and immunohistochemical staining of FAP expression were performed to present the tumor progression and difference on molecular level. In detail, the slides were incubated overnight with fibroblast activation protein (FAP) antibody (ab53066, rabbit polyclonal IgG: diluted 1:100; Abcam, Shanghai, China) at 4℃. Then, slices were incubated with a secondary goat anti-rabbit antibody (cat. no. GB23303; Wuhan Servicebio Technology Co., Ltd., Shanghai, China; 1:200) at room temperature for 50 min. FAP expression was quantified with Image for the staining intensity of FAP-positive area.

### 4.9. Statistics

The differences of tracer uptake between FAP-positive and FAP-negative models were evaluated with independent samples *t*-test, and any difference with *p*-value < 0.05 was statistically significant.

## 5. Conclusions

^99m^Tc-HYNIC-FAPI-04 extended the differential time window in evaluating tumor fibrosis, as well as intensified the advantage of high target-to-background ratio.

## Data Availability

Data is contained within the article.
